# First person – Sarah Woodfield

**DOI:** 10.1242/bio.059367

**Published:** 2022-09-13

## Abstract

First Person is a series of interviews with the first authors of a selection of papers published in Biology Open, helping early-career researchers promote themselves alongside their papers. Sarah Woodfield is first author on ‘ HepT1-derived murine models of high-risk hepatoblastoma display vascular invasion, metastasis, and circulating tumor cells’, published in BiO. Sarah is assistant professor of surgery at Baylor College of Medicine and the Texas Children's Hospital in Houston, investigating the high-risk features of hepatoblastoma, including primarily metastasis and chemoresistance, with the overall goal of better understanding these characteristics in order to target them with novel inhibitors to improve patient outcomes.


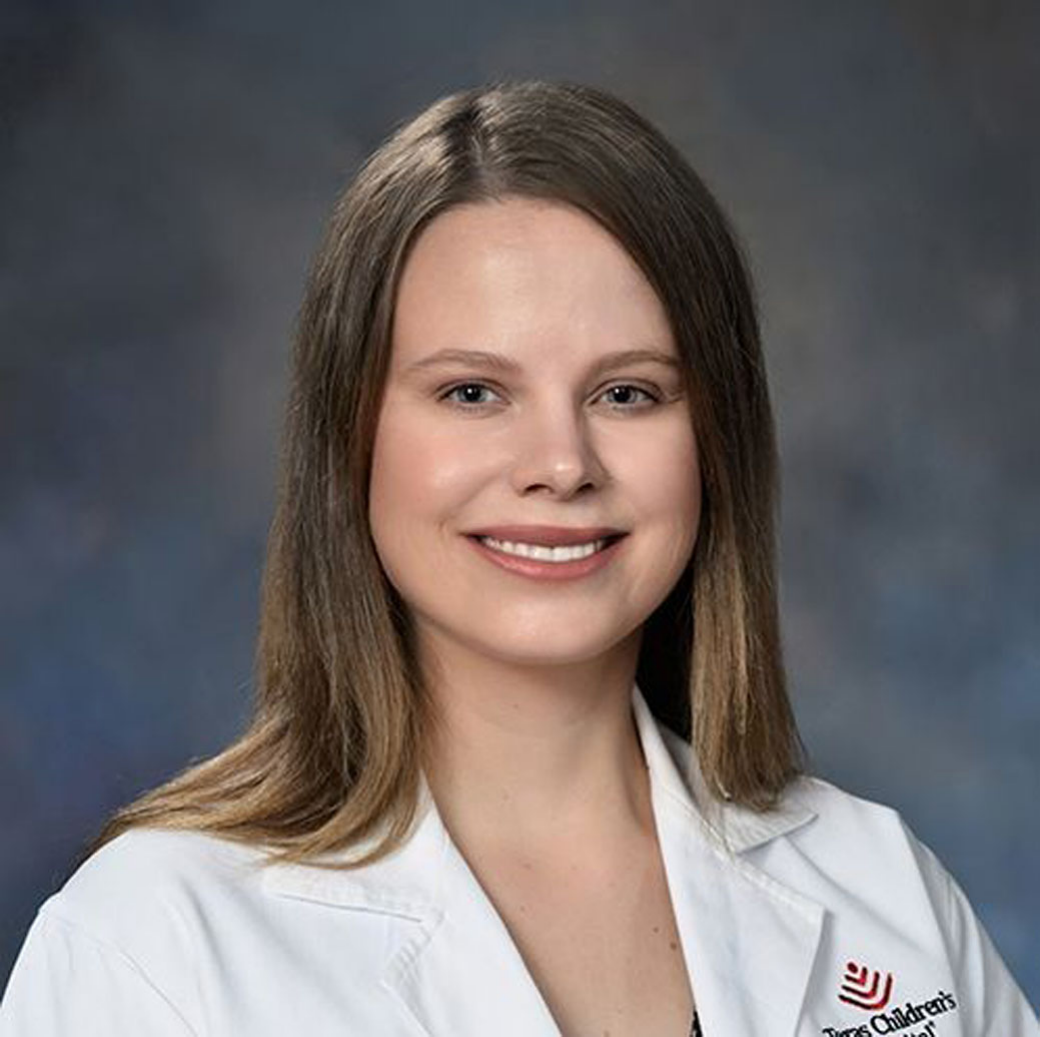


**Sarah Woodfield**

**What is your scientific background and the general focus of your lab?**

I completed my PhD in developmental biology, and my thesis work focused on characterizing a model of neoplastic tumorigenesis in the fruit fly, *Drosophila melanogaster*. With this background in developmental biology, I became very interested in pediatric cancer, which generally results from a failure of normal mechanisms of development to occur. After graduate school, I focused my postdoctoral research effort on two forms of pediatric cancer, neuroblastoma and hepatoblastoma. Hepatoblastoma is the most common primary tumor of the liver seen in children and has the fastest rising incidence of all pediatric solid tumors. As a postdoctoral fellow, I developed and characterized novel mouse and cell-line models of hepatoblastoma and tested various targeted therapies with these models. More recently, my independent lab studies phenotypes of high-risk hepatoblastoma, including primarily metastasis and chemoresistance. We are primarily interested in studying circulating tumor cells, including characterizing these unique cells and elucidating ways to target these cells with novel drugs. We are also working to develop and test a liquid biopsy assay for circulating tumor cells for hepatoblastoma patients, which would represent a clear improvement to the current standard of care for disease surveillance.

**How would you explain the main findings of your paper to non-scientific family and friends?**

In this paper, we develop and thoroughly characterize several novel mouse models of hepatoblastoma. These models are generated with injection of human cells into either the mouse livers or into the mouse tails. Characteristics of high-risk disease are replicated by these models. In the mice, large tumors composed of human cells grow in the livers and in the lungs, and the lung tumors represent metastatic disease. In addition, human cells circulating in the mouse blood, representing circulating tumor cells, are found. Finally, we looked at expression of genes in different tumor areas, including tumor cells from the livers and lungs and cells inside vessels. We compared expression from these various samples to show changes in gene expression that may contribute to the aggressive movement of cells from the livers through the whole blood and into the lungs.

**What are the potential implications of these results for your field of research?**

Hepatoblastoma is a very rare tumor and, thus, there are few models of disease that can be used in the laboratory setting for meaningful preclinical studies. These models represent new murine models that show high-risk phenotypes, such as vascular invasion and metastasis, that can be used for relevant studies. As we did in this work with mRNA sequencing, profiling the different compartments of disease may shed light on how cells change in order to invade vessels, migrate, and seed metastatic disease. More importantly, testing of novel targeted agents with these models may show whether these drugs inhibit specific high-risk features, such as metastasis. Finally, this work shows that implantation technique of otherwise identical cells influences how they implant and grow in a mouse and may influence future model development. In this study, when we injected HepT1 cells into the liver, they gave rise to large intrahepatic tumors with lung metastasis. On the other hand, when we forced them into circulation by injecting them into the tail vein, we saw liver tumors and higher incidence of lung metastasis.

“Experiments fail so often that it is absolutely essential to celebrate every success.”

**Figure:**
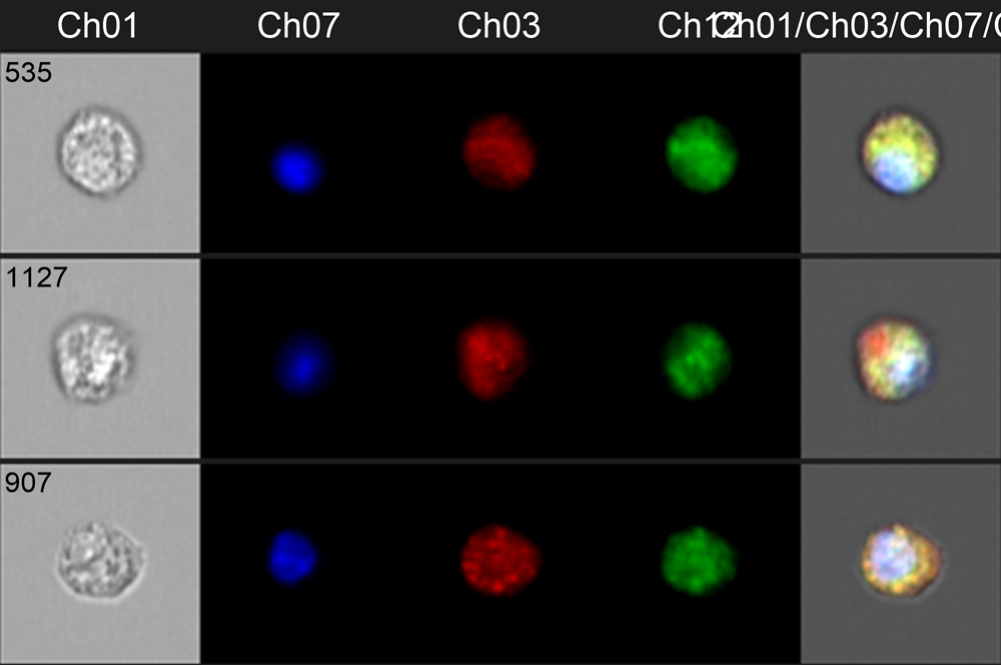
Primary circulating tumor cells isolated from the whole blood of a patient with hepatoblastoma.

**What has surprised you the most while conducting your research?**

What has surprised me the most while conducting research is how much attitude influences success. Experiments fail so often that it is absolutely essential to celebrate every success. I've worked with individuals who have years of training and have not been very successful because they do not have positive attitudes, they do not persevere, and they do not embrace the research experience. On the other hand, I've worked with very inexperienced undergraduates that have very positive attitudes and are very hard working, who have generated very beautiful data and have contributed to multiple publications. I feel like I can teach someone how to think about and conduct successful experiments but I cannot teach someone how to have a positive attitude appropriate to the ups and downs of academic laboratory research.

**What, in your opinion, are some of the greatest achievements in your field and how has this influenced your research?**

The field of cancer research is vast, and, thus, it is hard to answer that question. My favorite paper is the classic Hanahan and Weinberg paper about the hallmarks of cancer. In my opinion, this paper changed the way we think about tumor cells and unified the field by looking at common characteristics that we see in tumor cells from widely different tumor origins. The field of hepatoblastoma, compared to work with other adult tumors, is relatively immature. That is one reason that I love working in this area; I feel that the field is not saturated and that I have the real opportunity to affect how this tumor is understood and treated. In this field, the work from Cairo et al., (2008) and Sumazin et al., (2017) showed that in this tumor, which has relatively few mutations, changes in gene expression are major contributors to disease. This work influenced our choice to use transcriptomic sequencing in this paper to look at changes in gene expression in the different tumor compartments.

**What changes do you think could improve the professional lives of early-career scientists?**

In the career journey of a scientist, through graduate school to postdoctoral positions and ultimately to an academic principal investigator, we receive relatively little training on how to be a successful manager, boss, and mentor. I have worked hard more recently to try to find opportunities to improve myself in these areas, and I think more training in these areas would be helpful. Sometimes, I feel like I just woke up one morning and suddenly went from being a mentee to needing to lead a lab. I also think that academic principal investigators wear too many hats and there is never enough time. I try to force myself to reflect on the big picture of what we are doing in the lab and to keep up with the literature because I think otherwise we lose sight of our overall goals.

“Sometimes, I feel like I just woke up one morning and suddenly went from being a mentee to needing to lead a lab.”

**What's next for you?**

I am now an early-career principal investigator. My lab is currently funded by a Department of Defense Career Development Award. My next major goal is to land a larger grant such as an R01 in order to really push our work forward. I am also a mother to two young girls. I hope that this year I can find more stability in my life and really feel like I am balancing my personal and professional spheres in an appropriate way.
